# Temporal Physiologic Profiles Associated with Return of Spontaneous Circulation in a Swine Model of Cardiopulmonary Resuscitation

**DOI:** 10.3390/jcm15176682

**Published:** 2026-08-28

**Authors:** Barbara Fyntanidou, Eirini Oloktsidou, Katerina Kotzampassi, Marios G. Bantidos, Christos Kofos, Andreas S. Papazoglou, Georgios Kazakos, Aikaterini Apostolopoulou, Athina Nasoufidou, Alexandra Arvanitaki, Maria Fasoula, Efstratios Karagiannidis, Vasilios Grosomanidis

**Affiliations:** 1 Department of Emergency Medicine, AHEPA University General Hospital, School of Medicine, Aristotle University of Thessaloniki, Stilponos Kyriakidi 1, 54636 Thessaloniki, Greece; bfyntan@yahoo.com (B.F.); katapost@yahoo.gr (A.A.); m.occludent@gmail.com (M.F.); 2 Department of Anesthesiology and Critical Care, AHEPA University General Hospital, School of Medicine, Aristotle University of Thessaloniki, Stilponos Kyriakidi 1, 54636 Thessaloniki, Greece; eirini_naki@yahoo.gr (E.O.); vgrosoma@auth.gr (V.G.); 3 Department of Surgery, Aristotle University of Thessaloniki, 54636 Thessaloniki, Greece; kakothe@yahoo.com; 4 Second Department of Cardiology, Hippokration General Hospital, School of Medicine, Aristotle University of Thessaloniki, Konstantinoupoleos 49, 54642 Thessaloniki, Greece; chriskofos21@gmail.com (C.K.); alexandra.arvanit@gmail.com (A.A.); 5 Athens Naval Hospital, Deinokratous 70, 11521 Athens, Greece; anpapazoglou@yahoo.com; 6 Anesthesia and Intensive Care Unit, Companion Animal Clinic, School of Veterinary Medicine, Aristotle University of Thessaloniki, Stavrou Voutyra 11, 54627 Thessaloniki, Greece; gkdvm@vet.auth.gr

**Keywords:** animal study, cardiac arrest, cardiopulmonary resuscitation, hemodynamics, multimodal monitoring, return of spontaneous circulation

## Abstract

**Background:** Physiology-guided cardiopulmonary resuscitation (CPR) is increasingly recognized as a strategy to move beyond mechanical targets, but evidence remains limited and dominated by isolated metrics. We characterized multimodal physiologic profiles associated with return of spontaneous circulation (ROSC) in a swine model. **Methods:** We analyzed 24 swine undergoing standardized cardiac arrest followed by CPR. Variables included end-tidal carbon dioxide (ETCO_2_), systolic arterial pressure (SAP), diastolic arterial pressure (DAP), right ventricular systolic pressure (RVSP), carotid artery blood flow, stroke volume, and cerebral regional oxygen saturation. The primary early analysis used per-animal medians from minutes 1–5 of CPR, before any animal achieved ROSC. Later prespecified 5 min windows were evaluated exploratorily because they increasingly included post-ROSC measurements. A pre-ROSC sensitivity analysis, correlation analysis, and exploratory principal component analysis (PCA) with bootstrap assessment of loading stability were also performed. **Results:** Nine animals (37.5%) achieved ROSC, with a median time to ROSC of 10 min (range 6–18 min). During minutes 1–5, before any ROSC occurred, animals subsequently achieving ROSC had higher carotid flow, DAP, ETCO_2_, right ventricular pressure signal, and SAP; all differences remained significant after false discovery rate correction. In the pre-ROSC sensitivity analysis, ETCO_2_, SAP, DAP, right ventricular pressure signal, carotid flow, and stroke volume differed between animals subsequently achieving ROSC and those that did not (all *p* < 0.001), whereas cerebral regional oxygen saturation did not (*p* = 0.474). Later analytical windows increasingly included post-ROSC measurements and were therefore interpreted exploratorily. PCA summarized the shared early physiologic variation, with PC1 explaining 69.8% of variance; bootstrap analysis showed consistent positive contributions of the included variables to PC1. **Conclusions:** Successful resuscitation was associated with a distinct pressure–flow–gas-exchange profile during early and pre-ROSC CPR. These findings support further investigation of multimodal physiology-guided resuscitation.

## 1. Introduction

Cardiac arrest remains a major cause of mortality despite advances in healthcare systems, algorithm-based cardiopulmonary resuscitation (CPR), and post-arrest management [1,2]. Current guidelines emphasize high-quality CPR, early defibrillation when indicated, and timely vasoactive therapy [2].

The rationale behind physiologic monitoring during CPR stems from the ability to assess the biologic effect of resuscitative efforts rather than simply the mechanics of CPR delivery. Current evidence appears dispersed across limited clinical and diverse experimental work [3,4]. Successful return of spontaneous circulation (ROSC) depends on adequate myocardial and systemic perfusion, with coronary perfusion pressure (CPP) recognized as a key hemodynamic correlate of resuscitation success [5,6]. End-tidal carbon dioxide (ETCO_2_) has been widely used as a practical surrogate of pulmonary blood flow and cardiac output during low-flow states, while invasive arterial pressure provides real-time information about compression-generated perfusion [7,8].

At the same time, interest has expanded beyond single hemodynamic variables. Multimodal monitoring approaches incorporating ETCO_2_, arterial blood pressure, near-infrared spectroscopy (NIRS), Doppler-derived flow signals, and echocardiographic assessment have emerged as potentially complementary tools during CPR [9,10].

However, most studies have examined individual markers in isolation or focused on a limited portion of the resuscitation period. In light of this, the present study examined the relationship between multiple early intra-arrest physiologic signals and subsequent ROSC in a swine cardiac arrest model, while additionally exploring their temporal behavior and shared multivariable structure.

## 2. Methods

### 2.1. Study Design and Outcome Definition

This study analyzed data collected from an experimental dataset of 24 swine subjected to standardized cardiac arrest followed by CPR. The primary outcome was ROSC, defined as the restoration of sustained, spontaneous arterial pulsatility. For animals achieving ROSC, the time to ROSC was recorded in minutes from the initiation of CPR. The study was conducted and reported in accordance with the ARRIVE 2.0 guidelines (Animal Research: Reporting of In Vivo Experiments) [11]. The original experimental protocol included an a priori sample size calculation using G*Power 3.1.7. A total sample of 22 animals was estimated to provide 80% power at α = 0.05, based on pilot ETCO_2_ measurements and a prespecified clinically relevant change. The available experimental cohort comprised 24 animals.

### 2.2. Ethical Considerations

All experimental procedures were performed in accordance with institutional guidelines and national regulations governing the care and use of laboratory animals, and were approved by the Directorate of Veterinary Services, Region of Central Macedonia, Greece (protocol no. 107675/742; approval date: 3 April 2015).

### 2.3. Animal Characteristics and Preparation

Healthy juvenile male swine (approximately 3 months of age and weighing 25 kg) were included. All animals were anesthetized, endotracheally intubated, mechanically ventilated, and instrumented before induction of cardiac arrest. All efforts were made to minimize suffering while preserving scientific validity.

### 2.4. Cardiac Arrest Model and Resuscitation Protocol

Cardiac arrest was induced under general anesthesia using transvenous pacing in conjunction with interruption of ventilation. After 7 min of untreated cardiac arrest, CPR was initiated with mechanical chest compressions using the Lund University cardiopulmonary assist system (LUCAS) device (Jolife AB, Lund, Sweden) and controlled ventilation through an endotracheal tube with a Siemens Servo 900C ventilator (Siemens AG, Munich, Germany). The untreated arrest interval was selected to model a prolonged yet potentially reversible arrest, after the electrical phase and within the circulatory phase, consistent with prior conceptual and porcine resuscitation models [12,13]. After 5 min of basic life support, advanced life support was initiated, including rhythm assessment, defibrillation for shockable rhythms, and drug administration. Both basic and advanced life support were performed according to the 2015 European Resuscitation Council resuscitation guidelines. In animals without ROSC, resuscitation efforts continued for up to 40 min, whereas animals achieving ROSC were supported for 4 h and subsequently euthanized according to the experimental protocol using thiopental and potassium chloride. The experimental timeline, including the untreated arrest interval, initiation of BLS and ALS, analytical windows, and observed timing of ROSC, is summarized in Supplementary Figure S2.

### 2.5. Physiological Data Acquisition and Structure

Physiological variables were recorded at baseline, immediately after cardiac arrest, and subsequently at minute-level resolution throughout CPR. Recorded variables included ETCO_2_, systolic arterial pressure (SAP), diastolic arterial pressure (DAP), right ventricular (RV) systolic pressure (RVSP), carotid artery blood flow, stroke volume, and cerebral regional oxygen saturation (rSO_2_). Analyses of CPR physiology were based on per-animal summary measures to avoid pseudo-replication from repeated minute-level observations. RVSP measurements obtained during CPR were interpreted as pressure signals generated during mechanical resuscitation, acknowledging potential influences from compression mechanics, intrathoracic pressure transmission, loading conditions, and cardiovascular response.

Systemic arterial pressure was measured invasively through a femoral arterial catheter. Carotid blood flow was measured using a circumferential flow probe placed around the common carotid artery, cerebral rSO_2_ by near-infrared spectroscopy (INVOS), and ETCO_2_ by capnography connected to the endotracheal breathing circuit. Hemodynamic measurements were acquired using invasive monitoring, including pulmonary artery catheterization where applicable.

### 2.6. Statistical Analysis

#### 2.6.1. Baseline Comparisons

Continuous baseline variables were summarized as medians [Q1–Q3] and compared between ROSC and no-ROSC groups using Wilcoxon rank-sum tests because of the limited sample size and non-normal distributions.

#### 2.6.2. Time-Resolved Analysis

CPR was divided a priori into four non-overlapping 5 min windows: minutes 1–5, 6–10, 11–15, and 16–20. The 1–5 min interval constituted the primary early CPR window because no animal achieved ROSC before minute 6. For each animal, the median value of each physiologic variable was calculated within each available window and compared between outcome groups using Wilcoxon rank-sum tests. False discovery rate control was applied across variables and time windows using the Benjamini–Hochberg procedure. Because ROSC occurred during the later analytical intervals, the contribution of post-ROSC observations was assessed for each window. Animals achieving ROSC were classified according to whether they remained under active CPR or had achieved ROSC within each interval. Analyses of the 6–10, 11–15, and 16–20 min windows were therefore considered exploratory temporal analyses rather than pure intra-arrest comparisons. Jonckheere–Terpstra tests were retained as exploratory assessments of monotonic change across ordered windows.

#### 2.6.3. Pre-ROSC Sensitivity Analysis

To assess whether physiologic differences between outcome groups preceded restoration of spontaneous circulation, a pre-ROSC sensitivity analysis was performed. For animals achieving ROSC, all measurements obtained at or after the first documented ROSC minute were excluded. Animals without ROSC retained all available CPR measurements. For each animal, the median value of each physiologic variable during the available pre-ROSC period was calculated and compared between animals that subsequently achieved ROSC and those that did not, using Wilcoxon rank-sum tests.

#### 2.6.4. Correlation Analysis of Early CPR Physiology

Among physiologic variables demonstrating consistent associations with ROSC during early CPR, pairwise associations were assessed using Spearman’s rank correlation based on per-animal median values from the first CPR window (minutes 1–5). Correlation *p*-values were adjusted using Benjamini–Hochberg correction.

#### 2.6.5. Exploratory Principal Component Analysis

PCA was performed as an exploratory dimensionality-reduction approach to summarize shared variation among correlated early CPR physiologic variables rather than as a predictive model. Standardized per-animal median values from minutes 1–5 were used, ensuring that each animal contributed one multivariable observation before any ROSC occurred. Variables included ETCO_2_, SAP, DAP, RVSP, and carotid artery blood flow. PCA was performed using singular value decomposition after standardization, and the first two principal components were retained for visualization and interpretation. The previously specified permutation comparison of PC1 scores between outcome groups was interpreted as exploratory and not as statistically independent confirmation of the univariable comparisons, because PC1 was derived from the same underlying variables. Relative variable contributions were estimated from component loadings weighted by the variance explained by PC1 and PC2. To assess loading stability given the limited sample size, animals were additionally resampled with replacement over 1000 bootstrap iterations; PCA was repeated in each bootstrap sample, and mean PC1 loadings with 95% bootstrap confidence intervals were calculated.

#### 2.6.6. Statistical Software

Analyses were performed using R version 4.5.3 (R Core Team, R Foundation for Statistical Computing, Vienna, Austria) and Python version 3.14.5 (Python Software Foundation). Statistical significance was evaluated at the 0.05 level after correction for multiple testing when appropriate.

## 3. Results

### 3.1. Study Population

Of the 24 animals included in the analysis, 9 (37.5%) achieved ROSC and 15 (62.5%) did not. Time to ROSC among animals achieving ROSC ranged from 6 to 18 min, with a median of 10 min. Because ROSC occurred between minutes 6 and 18, later analytical windows increasingly included post-ROSC measurements among animals achieving ROSC. During minutes 1–5, all nine animals subsequently achieving ROSC remained under active CPR. During minutes 6–10, four remained under CPR and five had achieved ROSC; during minutes 11–15, three remained under CPR and six had achieved ROSC; and during minutes 16–20, all nine had achieved ROSC. The detailed composition of the analytical windows is provided in Supplementary Table S1.

### 3.2. Baseline Physiology

No statistically significant baseline differences were detected between outcome groups. Although the difference did not reach statistical significance, baseline carotid flow was numerically lower in animals that subsequently achieved ROSC (67 [65–112] vs. 109 [93.5–126.5] mL/min; *p* = 0.111); given the small sample size, this numerical imbalance should be acknowledged (Table 1).

### 3.3. Early CPR Physiology (Minutes 1–5)

During the first 5 min of CPR, before any animal had achieved ROSC, animals that subsequently achieved ROSC demonstrated higher ETCO_2_, SAP, DAP, RV pressure signal, and carotid flow than animals that did not achieve ROSC. All differences remained significant after FDR correction (Table 2), whereas cerebral rSO_2_ and stroke volume did not differ between groups.

### 3.4. Pre-ROSC Sensitivity Analysis

In the pre-ROSC sensitivity analysis, significant differences between animals that subsequently achieved ROSC and those that did not remained present for ETCO_2_, SAP, DAP, RV pressure signal, carotid flow, and stroke volume (all *p* < 0.001). Cerebral rSO_2_ remained non-discriminatory (*p* = 0.474). Carotid flow was available for 19 animals in this analysis, whereas the remaining variables included all 24 animals (Supplementary Table S2). These findings indicate that the principal physiological differences were already present before restoration of spontaneous circulation.

### 3.5. Exploratory Time-Resolved Physiology Across Later Analytical Windows

Between-group differences were also observed in later analytical windows (Table 2; Supplementary Figure S1). The magnitude of later-window separation should not be interpreted as evidence that intra-arrest physiological discrimination increased after the first 5 min, although these windows increasingly included measurements obtained after ROSC among animals achieving spontaneous circulation.

### 3.6. Exploratory Temporal Trajectories and Trend Analysis

**Figure 1:**
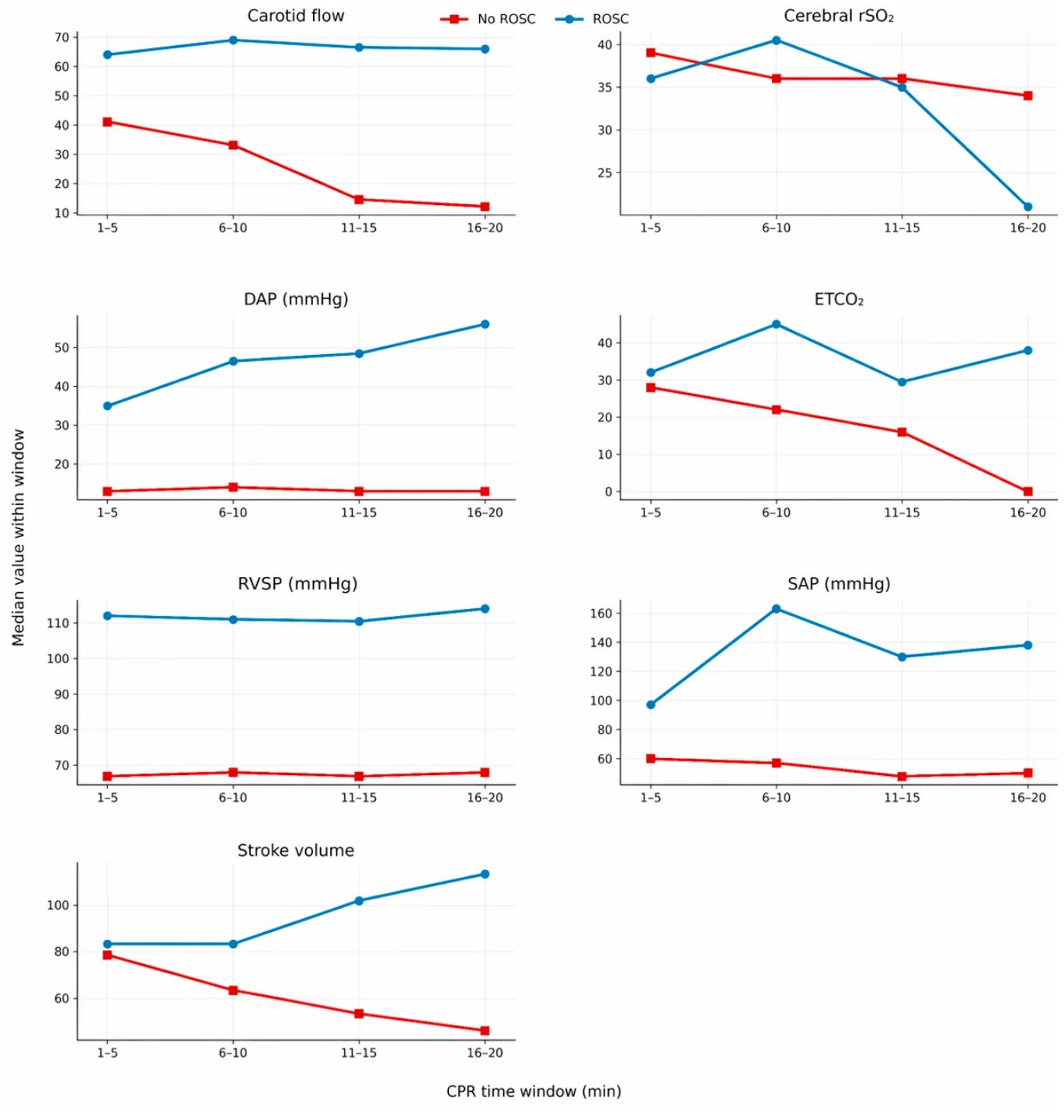
**Time-resolved physiologic trajectories during CPR stratified by ROSC status.** Median values of ETCO_2_, SAP, DAP, RVSP, carotid artery blood flow, stroke volume, and rSO_2_ are shown across predefined CPR windows (1–5, 6–10, 11–15, and 16–20 min). Values represent per-animal medians within each time window, plotted separately for animals achieving ROSC (blue circles) and animals not achieving ROSC (red squares). Later windows in the ROSC group increasingly include post-ROSC observations and should therefore be interpreted with caution. Abbreviations: CPR: cardiopulmonary resuscitation; DAP: diastolic arterial pressure; ETCO_2_: end-tidal carbon dioxide; ROSC: return of spontaneous circulation; RVSP: right ventricular systolic pressure; rSO_2_: regional oxygen saturation; RV: right ventricle; SAP: systolic arterial pressure.

Descriptive trajectories across the prespecified windows differed between outcome groups (Figure 1). Animals not achieving ROSC showed progressive declines in several physiologic measures during ongoing resuscitation. Jonckheere–Terpstra trend analyses are presented as exploratory, since later windows increasingly included post-ROSC observations (Table 3).

### 3.7. Correlation Structure of Early CPR Physiology

**Figure 2:**
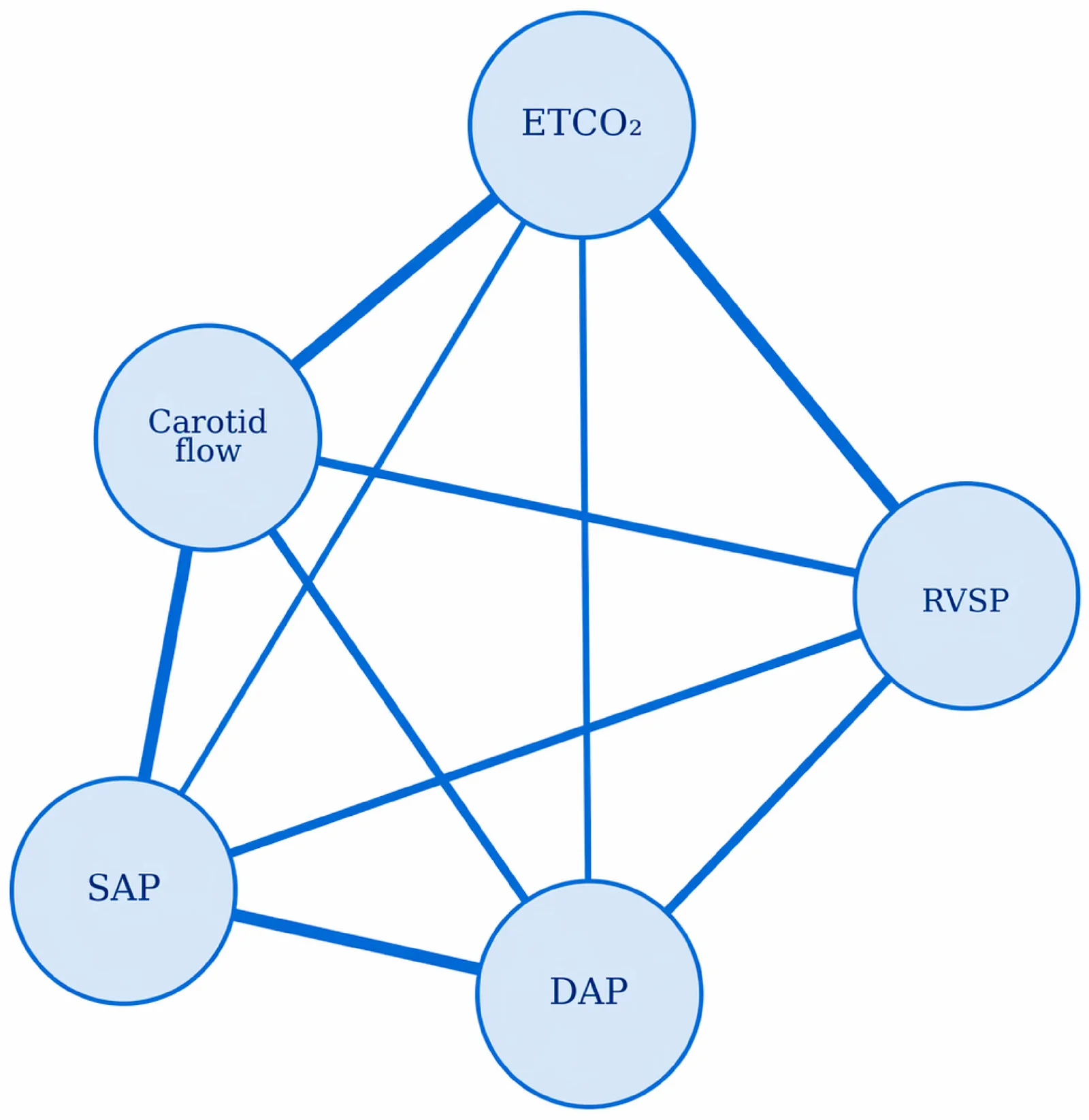
**Correlation structure of early CPR physiology.** Network representation of Spearman correlations among early CPR physiologic variables based on per-animal medians. Edge thickness reflects the relative strength of the positive correlations, with thicker lines indicating stronger associations. Abbreviations: CPR: cardiopulmonary resuscitation; DAP: diastolic arterial pressure; ETCO_2_: end-tidal carbon dioxide; RVSP: right ventricular systolic pressure; SAP: systolic arterial pressure.

During minutes 1–5, Spearman correlation analysis indicated positive associations among ETCO_2_, SAP, DAP, RVSP, and carotid flow (Figure 2), suggesting coordinated behavior among early resuscitation physiological signals.

### 3.8. Exploratory Multivariable Structure of Early CPR Physiology

**Figure 3:**
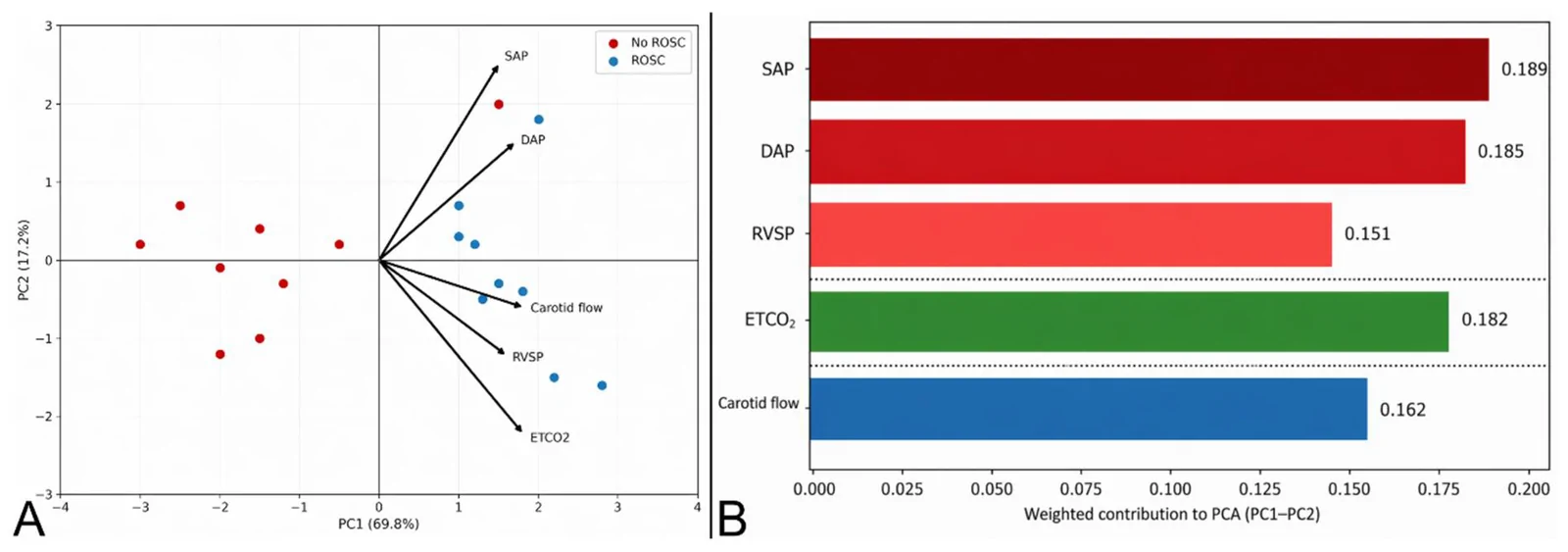
**Exploratory multivariable structure of early CPR physiology and relative variable contributions.** (**A**). Scatterplot of individual animals projected onto the first two principal components (PC1 and PC2) using standardized median values from the first 5 min of CPR. Points represent individual animals and are colored by outcome (ROSC, blue; no ROSC, red). Arrows indicate variable loadings; the arrow direction reflects increasing values of each variable, and the arrow length reflects the magnitude of the loading vector in the PC1-PC2 plane. PC1 explained 69.8% and PC2 17.2% of the total variance. (**B**). Relative contribution of each variable to the principal component structure. Variables are grouped by physiologic domain for visual clarity. Abbreviations: CPR: cardiopulmonary resuscitation; DAP: diastolic arterial pressure; ETCO_2_: end-tidal carbon dioxide; PC: principal component; PCA: principal component analysis; ROSC: return of spontaneous circulation; RVSP: right ventricular systolic pressure; SAP: systolic arterial pressure.

Exploratory PCA of standardized early CPR physiological medians identified a dominant first principal component (PC1) explaining 69.8% of the total variance, with PC1 and PC2 together accounting for 87.0% (Figure 3A). ETCO_2_, SAP, DAP, RV pressure signal, and carotid flow contributed positively to PC1, reflecting their shared covariance during early CPR. Animals subsequently achieving ROSC were separated from no-ROSC animals along PC1; however, because PC1 was derived from the same physiologic variables examined in the univariable analyses, this separation should not be interpreted as statistically independent validation of a distinct phenotype. Bootstrap analysis across 1000 resamples demonstrated consistently positive PC1 loadings for ETCO_2_, SAP, DAP, RV pressure signal, and carotid flow (Supplementary Table S3), supporting the internal stability of the loading pattern.

## 4. Discussion

### 4.1. Principal Findings

In this experimental model, animals subsequently achieving ROSC showed a distinct physiologic profile within the first 5 min of CPR, before ROSC occurred in any animal. Higher arterial pressures, carotid flow, ETCO_2_, and RV pressure signals characterized subsequent ROSC, and the pre-ROSC sensitivity analysis confirmed that these differences were already present during active resuscitation, while cerebral rSO_2_ remained non-discriminatory. Later temporal patterns suggested progressive deterioration in animals without ROSC and recovery or preservation of key physiologic signals in those achieving ROSC, although later windows increasingly included post-ROSC measurements. PCA further summarized the coordinated behavior of early pressure, flow, and gas-exchange variables, with bootstrap analysis supporting the stability of their shared loading pattern.

### 4.2. Mechanistic Interpretation

A mechanistic interpretation of these findings is that subsequent ROSC was associated with CPR generating not only higher pressures but also a more favorable compression–decompression cycle. Blood flow during CPR is produced by a variable combination of cardiac pump and thoracic pump mechanisms, and transesophageal echocardiographic work suggests that valve motion and intrathoracic pressure swings jointly determine whether compressions create useful forward flow or mostly pressure without effective circulation [14].

This matters because myocardial perfusion is largely a relaxation-phase phenomenon. Coronary flow during CPR depends on the diastolic aortic-to-right-atrial gradient, so a higher DAP is beneficial only if recoil and venous decompression prevent right atrial pressure from rising in parallel [5,15]. Experimental flow studies further show that coronary arterial flow is greatest during relaxation and can even reverse during the wrong phase, emphasizing that waveform quality and decompression likely matter as much as peak pressure itself [16].

Seen in that light, the higher SAP, DAP, RV pressure signal, ETCO_2_, and carotid flow in ROSC animals likely reflect a coordinated state in which compressions generated forward aortic flow, recoil preserved venous return, and pulmonary transit remained sufficient to maintain CO_2_ delivery to the lungs. The RV pressure signal recorded during mechanical CPR should not be interpreted as a conventional measure of intrinsic right ventricular systolic function; rather, it likely reflects a composite of compression mechanics, intrathoracic pressure transmission, loading conditions, and cardiovascular response. ETCO_2_ therefore probably tracked not simply “cardiac output” in a generic sense, but the efficiency of blood transit through the right heart, pulmonary circulation, and left-sided outflow [17]. By contrast, cerebral rSO_2_ may have been less discriminative early because it is influenced by downstream factors such as cerebral venous volume, oxygen extraction, and delayed microcirculatory recruitment rather than immediate central hemodynamics alone [18,19].

### 4.3. Comparison with the Prior Literature

The present findings are broadly consistent with the prior CPR physiology literature, but they also extend it in an important way. Earlier work established that pressure-based hemodynamics matter: the classic invasive human study by Paradis et al. linked higher CPP to ROSC, while porcine studies of hemodynamic-directed resuscitation by Friess et al. and Sutton et al. showed improved outcomes when CPR was guided by physiologic targets rather than by compression depth alone [5,8]. Clinical registry data from Sutton et al. further suggest that the use of physiologic monitoring during adult cardiac arrest is associated with higher ROSC [3]. Our data align with this body of work in showing that animals subsequently achieving ROSC had higher arterial pressures during the early intra-arrest period.

A similar comparison applies to ETCO_2_. Systematic reviews by Touma et al., Davies et al., and Paiva et al. support the association between higher ETCO_2_ and ROSC, while experimental work such as Hamrick et al. suggests that ETCO_2_ can even be used as a feedback target during CPR [7,17,20]. In our study, ETCO_2_ was higher during early CPR in animals subsequently achieving ROSC, and this association remained evident in the pre-ROSC sensitivity analysis.

The more novel comparison involves NIRS, carotid flow, and cardiac motion signals. Clinical studies by Engel et al. and Takegawa et al., together with the meta-analysis by Sanfilippo et al., suggest that cerebral oximetry may be useful during arrest, especially when trends rather than isolated values are considered [18,21,22]. Our results only partly align, since cerebral rSO_2_ was not an early discriminator, reinforcing the idea that NIRS may be more context-dependent and temporally buffered than central pressure or flow signals. By contrast, the early carotid flow findings were notable and complement the emerging Doppler literature, including the first-in-human feasibility study by Adedipe et al. and recent porcine continuous Doppler work by Mushunuri et al. [23,24].

Finally, although intra-arrest echocardiography has been described previously, markers such as RV systolic velocity and other tissue Doppler parameters were not assessed in the present study, as they are difficult to acquire reliably during CPR and would require an imaging-focused study protocol [25].

### 4.4. Strengths and Limitations

This study has several strengths. First, while much of the existing resuscitation literature has examined physiological variables in isolation, the present analysis integrates multiple complementary pressure, flow, gas-exchange, and oxygenation signals [26]. Second, the first analytical window captured a clearly defined intra-arrest period before ROSC occurred in any animal, while the pre-ROSC sensitivity analysis further assessed whether the observed differences preceded restoration of spontaneous circulation. Third, the controlled experimental setting reduced procedural heterogeneity and enabled simultaneous minute-level physiologic monitoring. Fourth, complementary analyses of individual signals, their correlation structure, temporal behavior, and shared multivariable variation provided a comprehensive characterization of resuscitation physiology. Finally, the variables examined retain translational relevance because several are already available, or increasingly feasible to obtain, in monitored resuscitation environments.

Several limitations should be considered. First, the sample size was modest, limiting generalizability. The identified physiologic pattern has not been evaluated in an independent experimental or clinical cohort and therefore requires external validation, although bootstrap resampling provided some assessment of its stability. Second, the animal model of healthy juvenile male swine under anesthesia and mechanical ventilation improved experimental consistency but did not reproduce the heterogeneity of human cardiac arrest, including older age, comorbid disease, diverse arrest etiologies, treatment delays, and variation in airway and ventilation conditions. In addition, although drug administration followed the standardized resuscitation protocol, individual-level timing and cumulative dose data were not available in a structured format suitable for reliable between-group analysis. Third, explicit calculation of CPP was not possible because usable right atrial pressure data were not available for the present analysis. DAP should not be interpreted as a direct surrogate for CPP. Fourth, later analytical windows increasingly included post-ROSC observations among successfully resuscitated animals, limiting their interpretation as purely intra-arrest physiology. Fifth, the variables included in the PCA represent related physiologic processes and are consequently correlated; PCA should therefore be regarded as a descriptive summary of shared physiologic variation rather than as validation of a distinct phenotype. Finally, baseline carotid flow was numerically lower in animals subsequently achieving ROSC, although the difference was not statistically significant; this finding should therefore be interpreted cautiously in light of the limited sample size.

### 4.5. Implications and Feasibility of Physiology-Guided CPR in Real-World Practice

Our findings support a physiology-guided CPR paradigm in which early resuscitation effectiveness is assessed through integrated pressure, flow, and gas-exchange signals. Current guidelines are broadly aligned with this direction, particularly in supporting waveform capnography and, when feasible, invasive arterial pressure monitoring during CPR [27]. However, translation into routine care will depend on more than physiological validity. Recent consensus work has identified major barriers to physiology-guided CPR, including limited usability outside critical care environments and the training and equipment required for monitoring. For instance, capnography uptake remains variable across institutions, with reported use ranging from 15% to 66% in emergency care settings, and access to capnography remains uneven across lower-resource settings [28].

Similar issues apply to newer modalities. Continuous carotid Doppler monitoring may allow confirmation of ROSC or optimization of chest compression position without interrupting compressions, but it requires dedicated equipment, operator familiarity, and integration into active workflows [10]. Future adequately powered studies should also evaluate the discriminative performance of individual and combined physiologic signals and determine whether reproducible, externally validated thresholds can be established for clinical use.

Physiology-guided CPR may initially need to be implemented in a tiered fashion, with simpler approaches used broadly and more advanced strategies reserved for higher-resource settings.

Financial considerations also matter: a 2024 cost-effectiveness analysis suggested that ETCO_2_ monitoring during in-hospital cardiac arrest was likely cost-effective even in a middle-income country setting [29]. Multimodal strategies will require similar evaluations to ensure value-based adoption.

## 5. Conclusions

Taken together, these findings suggest that early CPR physiology should be interpreted as an integrated response rather than as a collection of isolated values. In this swine model, subsequent ROSC was associated with a favorable pressure–flow–gas-exchange profile during early CPR, and key physiologic differences were also evident in the pre-ROSC sensitivity analysis. These findings provide a rationale for larger, externally validated studies testing whether multimodal physiologic monitoring can improve real-time assessment during CPR.

## Figures and Tables

**Table 1:** Baseline physiology by ROSC status.

Variable	No-ROSC (*n* = 15) Median [Q1–Q3]	ROSC (*n* = 9) Median [Q1–Q3]	*p*-Value
ETCO_2_ (mmHg)	47 [45.5–49.5]	51 [45–53]	0.491
Cerebral rSO_2_ (%)	55 [54–56]	51 [48–63]	0.570
RVSP (mmHg)	24 [22.5–26]	25 [23–26]	0.588
Stroke volume (mL)	80 [70–84.5]	85 [65–87]	0.454
DAP (mmHg)	72 [66.5–76]	82 [65–95]	0.698
SAP (mmHg)	116 [106.5–120]	115 [102–136]	0.881
Carotid flow (mL/min)	109 [93.5–126.5]	67 [65–112]	0.111

Abbreviations: DAP, diastolic arterial pressure; ETCO_2,_ end-tidal carbon dioxide; mmHg, millimeters of mercury; ROSC, return of spontaneous circulation; rSO_2,_ regional oxygen saturation; RVSP, right ventricular systolic pressure; SAP, systolic arterial pressure.

**Table 2:** Physiological variables by ROSC status across CPR time windows.

Variable	Window	ROSC Median [IQR]	No-ROSC Median [IQR]	FDR-Adjusted *p*-Value (q-Value)
Carotid flow (mL/min)	1–5 min	64 [10]	41 [24.5]	0.0047
	6–10 min	69 [4.4]	33 [30]	0.0015
	11–15 min	66.5 [6]	14.5 [18.5]	0.0078
	16–20 min	66 [6]	12 [13.9]	0.0147
DAP (mmHg)	1–5 min	35 [2]	13 [7]	0.0056
	6–10 min	46.5 [27.1]	14 [10]	0.0015
	11–15 min	48.5 [11.2]	13 [6]	0.0078
	16–20 min	56 [25.2]	13 [8]	0.0106
ETCO_2_ (mmHg)	1–5 min	32 [11]	28 [8.5]	0.0056
	6–10 min	45 [18.5]	22 [6.5]	0.0014
	11–15 min	29.5 [8.1]	16 [22]	0.0057
	16–20 min	38 [6.5]	0 [15]	0.0065
RVSP (mmHg)	1–5 min	112 [11]	67 [23]	0.0014
	6–10 min	111 [3]	68 [17]	0.0014
	11–15 min	110.5 [9]	67 [19]	0.0062
	16–20 min	114 [5]	68 [13]	0.0106
SAP (mmHg)	1–5 min	97 [10]	60 [16]	0.0036
	6–10 min	163 [49.5]	57 [23]	0.0014
	11–15 min	130 [13]	48 [19]	0.0119
	16–20 min	137.5 [25]	50 [21]	0.0178

Values are presented as median [IQR], where IQR denotes the interquartile range (Q3–Q1). Abbreviations: DAP: diastolic arterial pressure; ETCO_2_: end-tidal carbon dioxide; FDR: false discovery rate; IQR: interquartile range; ROSC: return of spontaneous circulation; RVSP: right ventricular systolic pressure; SAP: systolic arterial pressure.

**Table 3:** Jonckheere–Terpstra trend tests across CPR windows by outcome groups.

Variable	ROSC Status	Trend	*p*-Value
Carotid flow	No-ROSC	Decreasing	<0.001
	ROSC	No significant monotonic trend	0.359
Cerebral rSO_2_	No-ROSC	No significant monotonic trend	0.080
	ROSC	No significant monotonic trend	0.790
DAP	No-ROSC	No significant monotonic trend	0.616
	ROSC	Increasing	<0.001
ETCO_2_	No-ROSC	Decreasing	<0.001
	ROSC	No significant monotonic trend	0.639
RVSP	No-ROSC	No significant monotonic trend	0.488
	ROSC	No significant monotonic trend	0.639
SAP	No-ROSC	Decreasing	0.011
	ROSC	Increasing	0.044
Stroke volume	No-ROSC	Decreasing	<0.001
	ROSC	Increasing	0.034

Abbreviations: DAP, diastolic arterial pressure; ETCO_2,_ end-tidal carbon dioxide; ROSC, return of spontaneous circulation; rSO_2,_ regional oxygen saturation; RVSP, right ventricular systolic pressure; SAP, systolic arterial pressure.

## Data Availability

The data presented in this study are available from the corresponding author upon reasonable request. The data are not publicly available because the underlying experimental records are maintained in a non-public institutional archive and require additional curation before external sharing.

## Supplementary Materials

The following supporting information can be downloaded at: https://www.mdpi.com/article/10.3390/jcm15176682/s1, Figure S1. Heatmap of Benjamini–Hochberg-adjusted p-values (false discovery rate q-values) for comparisons between ROSC and no-ROSC animals across predefined CPR time windows. Color intensity represents −log10(q), with darker shading indicating stronger between-group separation; the displayed values are the corresponding q-values. The 1–5-min window preceded ROSC in all animals. Later windows increasingly included post-ROSC observations in the ROSC group and should therefore be interpreted cautiously, as between-group differences may partly reflect post-ROSC physiology. Figure S2. Schematic experimental timeline. Cardiac arrest was induced under general anesthesia by transvenous pacing with interruption of ventilation, followed by 7 min of untreated arrest. CPR was then initiated with mechanical chest compressions using the LUCAS device and controlled ventilation using a Siemens Servo 900C ventilator. Advanced life support began after 5 min of basic life support and included rhythm assessment, defibrillation for shockable rhythms, and drug administration according to the 2015 European Resuscitation Council guidelines. The prespecified analytical windows were minutes 1–5, 6–10, 11–15, and 16–20 after CPR initiation. Among animals achieving ROSC, ROSC occurred between 6 and 18 min (median 10 min). Table S1. Composition of analytical windows according to CPR status and ROSC timing. Table S2. Pre-ROSC sensitivity analysis of physiologic variables associated with subsequent ROSC. Table S3. Bootstrap stability analysis of PC1 loadings from the exploratory principal component analysis.

## Abbreviations

ARRIVE: Animal Research: Reporting of In Vivo Experiments; CPP: coronary perfusion pressure; CPR: cardiopulmonary resuscitation; DAP: diastolic arterial pressure; ETCO_2_: end-tidal carbon dioxide; FDR: false discovery rate; IQR: interquartile range; LUCAS: Lund University cardiopulmonary assist system; mmHg: millimeters of mercury; NIRS: near-infrared spectroscopy; PC: principal component; PC1: first principal component; PC2: second principal component; PCA: principal component analysis; ROSC: return of spontaneous circulation; rSO_2_: regional oxygen saturation; RVSP: right ventricular systolic pressure; SAP: systolic arterial pressure.
