# Supplementary material for: Temporal Physiologic Profiles Associated with Return of Spontaneous Circulation in a Swine Model of Cardiopulmonary Resuscitation
Source: J Clin Med. 2026 Aug 28;15(17):6682. doi: 10.3390/jcm15176682 (PMC13566323; doi:10.3390/jcm15176682)
Supplement: Supplementary file 1 [file jcm-15-06682-s001.zip › jcm-4472075-supplementary.pdf]

Supplementary Materials

Temporal Physiologic Profiles Associated with Return of Spontaneous Circulation in a Swine Model of Cardiopulmonary Resuscitation

Corresponding authors: Marios G. Bantidos (mbadidos@gmail.com) ; Efstratios Karagiannidis (stratoskarag@gmail.com)

Supplementary Table S1. Composition of analytical windows according to CPR status and ROSC timing.

CPR time window	ROSC-group animals still receiving CPR, n	ROSC-group animals with ROSC already achieved, n	No-ROSC animals, n
1–5 min	9	0	15
6–10 min	4	5	15
11–15 min	3	6	15
16–20 min	0	9	15

Note: ROSC occurred between 6 and 18 minutes (median 10 minutes). All animals in the no-ROSC group remained under active resuscitation during the analyzed windows.

Supplementary Table S2. Pre-ROSC sensitivity analysis of physiologic variables associated with subsequent ROSC.

Variable	N	p-value
$ETCO_2$	24	<0.001
SAP	24	<0.001
DAP	24	<0.001
RV pressure signal (RVSP)	24	<0.001
Carotid flow	19	<0.001
Stroke volume	24	<0.001
Cerebral rSO_2	24	0.474

Note: For animals achieving ROSC, measurements obtained at or after the first documented ROSC minute were excluded; animals without ROSC retained all available CPR measurements. Per-animal median pre-ROSC values were compared between outcome groups using Wilcoxon rank-sum tests. Carotid flow was available for 19 animals.

Supplementary Table S3. Bootstrap stability analysis of PC1 loadings from the exploratory principal component analysis.

Variable	Mean PC1 loading	95% bootstrap CI
$ETCO_2$	0.406	0.203–0.577
SAP	0.422	0.238–0.625
DAP	0.483	0.365–0.618
RV pressure signal (RVSP)	0.442	0.268–0.553
Carotid flow	0.437	0.252–0.586

Note: Animals were resampled with replacement over 1,000 bootstrap iterations. PCA was repeated in each bootstrap sample using standardized early CPR values, and mean PC1 loadings with 95% bootstrap confidence intervals were calculated. RVSP denotes the measured right ventricular systolic pressure variable, interpreted during mechanical CPR as an RV pressure signal rather than a conventional measure of intrinsic RV systolic function.

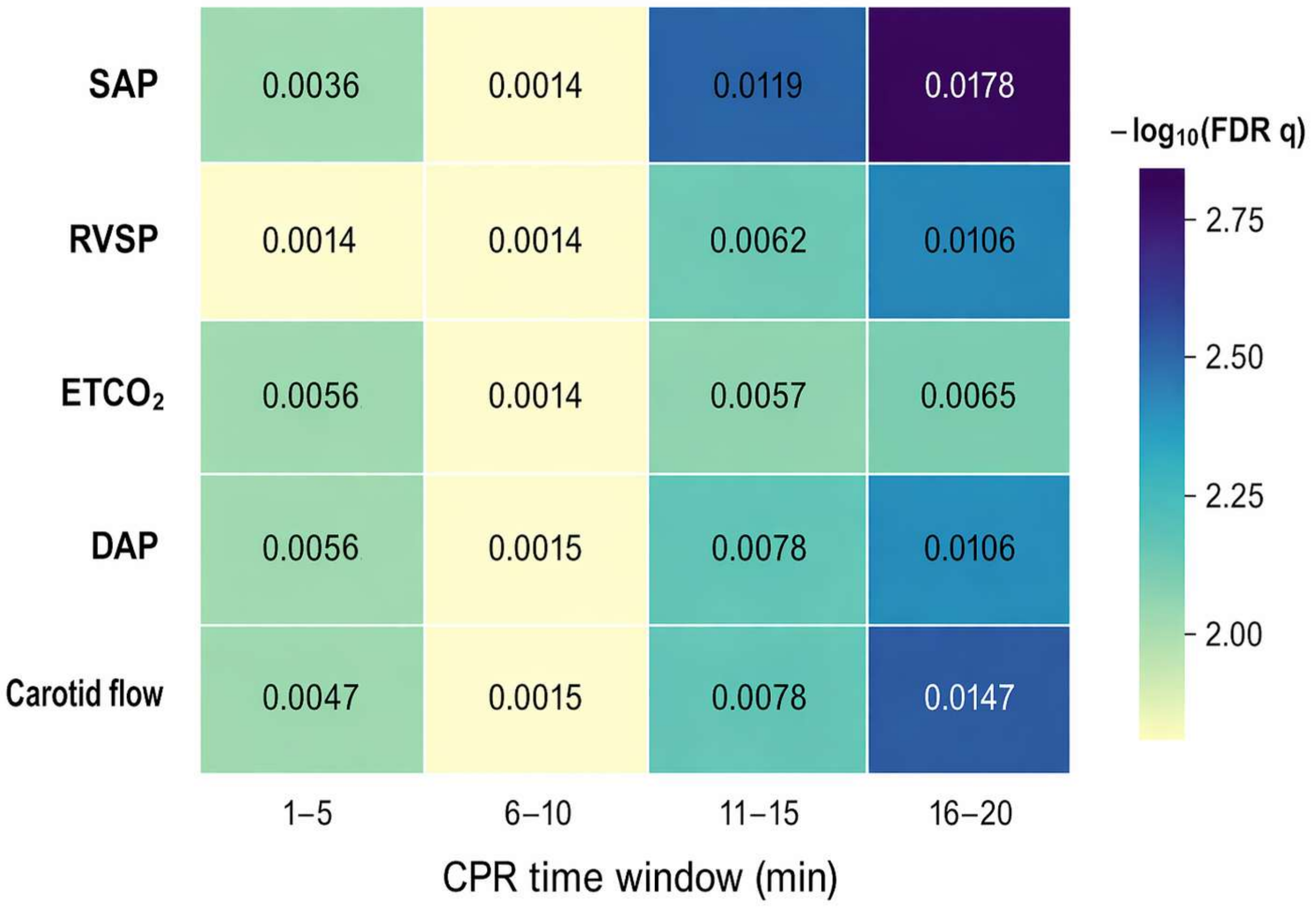

Supplementary Figure S1. Heatmap of Benjamini–Hochberg-adjusted p-values (false discovery rate q-values) for comparisons between ROSC and no-ROSC animals across predefined CPR time windows. Color intensity represents

−log10(q), with darker shading indicating stronger between-group separation; the displayed values are the corresponding q-values. The 1–5-minute window preceded ROSC in all animals. Later windows increasingly included post-ROSC observations in the ROSC group and should therefore be interpreted cautiously, as between-group differences may partly reflect post-ROSC physiology.

Abbreviations: CPR, cardiopulmonary resuscitation; DAP, diastolic arterial pressure; $ETCO_2$, end-tidal carbon dioxide; FDR, false discovery rate; q, adjusted p-value; ROSC, return of spontaneous circulation; RVSP, right ventricular systolic pressure; SAP, systolic arterial pressure.

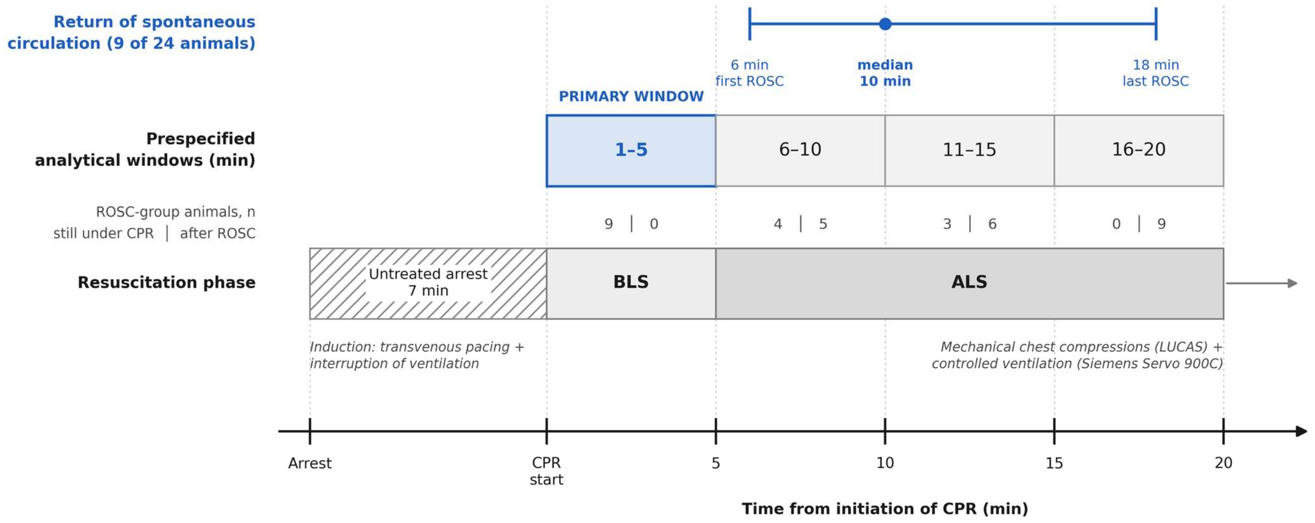

Supplementary Figure S2. Schematic experimental timeline. Cardiac arrest was induced under general anesthesia by transvenous pacing with interruption of ventilation, followed by 7 minutes of untreated arrest. CPR was then initiated with mechanical chest compressions using the LUCAS device and controlled ventilation using a Siemens Servo 900C ventilator. Advanced life support began after 5 minutes of basic life support and included rhythm assessment, defibrillation for shockable rhythms, and drug administration according to the 2015 European Resuscitation Council guidelines. The prespecified analytical windows were minutes 1–5, 6–10, 11–15, and 16–20 after CPR initiation. Among animals achieving ROSC, ROSC occurred between 6 and 18 minutes (median 10 minutes).

Abbreviations: ALS, advanced life support; BLS, basic life support; CPR, cardiopulmonary resuscitation; ERC, European Resuscitation Council; LUCAS, Lund University cardiopulmonary assist system; ROSC, return of spontaneous circulation.
